# Cupincin: A Unique Protease Purified from Rice (*Oryza sativa* L.) Bran Is a New Member of the Cupin Superfamily

**DOI:** 10.1371/journal.pone.0152819

**Published:** 2016-04-11

**Authors:** Roopesh Sreedhar, Purnima Kaul Tiku

**Affiliations:** Department of Protein Chemistry and Technology, CSIR-Central Food Technological Research Institute, Mysuru, Karnataka, India; Universidade Federal do Rio Grande do Sul, BRAZIL

## Abstract

Cupin superfamily is one of the most diverse super families. This study reports the purification and characterization of a novel cupin domain containing protease from rice bran for the first time. Hypothetical protein OsI_13867 was identified and named as cupincin. Cupincin was purified to 4.4 folds with a recovery of 4.9%. Cupincin had an optimum pH and temperature of pH 4.0 and 60°C respectively. Cupincin was found to be a homotrimer, consisting of three distinct subunits with apparent molecular masses of 33.45 kDa, 22.35 kDa and 16.67 kDa as determined by MALDI-TOF, whereas it eluted as a single unit with an apparent molecular mass of 135.33 ± 3.52 kDa in analytical gel filtration and migrated as a single band in native page, suggesting its homogeneity. Sequence identity of cupincin was deduced by determining the amino-terminal sequence of the polypeptide chains and by and *de novo* sequencing. For understanding the hydrolysing mechanism of cupincin, its three-dimensional model was developed. Structural analysis indicated that cupincin contains His313, His326 and Glu318 with zinc ion as the putative active site residues, inhibition of enzyme activity by 1,10-phenanthroline and atomic absorption spectroscopy confirmed the presence of zinc ion. The cleavage specificity of cupincin towards oxidized B-chain of insulin was highly specific; cleaving at the Leu_15_-Tyr_16_ position, the specificity was also determined using neurotensin as a substrate, where it cleaved only at the Glu_1_-Tyr_2_ position. Limited proteolysis of the protease suggests a specific function for cupincin. These results demonstrated cupincin as a completely new protease.

## Introduction

Proteases are chiefly regarded as protein-degrading enzymes. Currently, proteases are also considered as crucial signaling molecules having its implications in numerous vital processes [1]. Proteases comprise of about 60% of the total worldwide sale of enzymes and are being extensively used in food, pharmaceutical and detergent industries [2]. Proteases from plant sources have received particular interest because of their ability to remain active over a wide range of pH and temperature [3].

Proteases play key roles in plants by chipping into processes such as maturation or destruction of specific sets of proteins, in response to variations in environmental conditions and developmental procedures [4]. The structural and functional importance of proteolytic processing like enzyme activation is eminent. The proteolytic processing of protein precursors is a well-known example of site-specific limited proteolysis responsible for the production of mature proteins. A site-specific limited proteolysis, which resembles proteolytic processing, occurs in plants when the degradation of storage globulins starts during seed germination [5].

The enormous consideration for proteases, in addition to their specificity of their action, has attracted extensive attention in an attempt to exploit their physiological and biotechnological applications [6, 7]. The diverse selectivity of the proteases has been extensively exploited in different analytical applications where protease-like trypsin is applied in mass spectrometry analysis and N-terminal sequencing and proteases like thrombin or enterokinase are normally used in biotechnological applications such as removal of the tags from the recombinant proteins [1]. Conformational characteristics of proteins can be effectively investigated by limited proteolysis experiments. The approach relies on the fact that the sites of limited proteolysis along a polypeptide chain are characterised by enhanced backbone flexibility and therefore, proteolytic probes can pinpoint the sites of local unfolding in a protein chain [8]. Thus, there is a continuous quest for proteases with limited proteolysis.

India is the second largest producer of rice in the world, with annual production exceeding 152 million metric tons (FAOSTAT, 2012, http://faostat.fao.org/). Rice bran is a by-product of rice milling industries. Very few studies have been carried out on proteolytic enzymes in rice (*Oryza sativa* L.). A typical aspartic protease “oryzasin” was purified from the rice seed and had been characterized in detail [9–11]. Recently in rice, an atypical aspartic protease was identified as playing a major role in regulating *indica–japonica* hybrid sterility by conditioning embryo-sac fertility [12]. Chen et al., [13] have also reported on the systematic identification and phylogenetic analysis of rice aspartate protease (OsAP). More recently an aspartate protease purified from rice (OsAP65) was shown to be essential for rice pollen germination and growth [14]. However, studies on the cupin domain containing protease from any source have never been reported earlier.

The cupin superfamily is among the most diverse of any protein family described to date, which includes both enzymatic and non-enzymatic members. Though diverse in functions, amino acid sequences and domain organization, a common feature that allowed the unification in the cupin superfamily of proteins, was the presence of a β-barrel containing the nodular sequence motifs in their structure [15]. In the Pfam database 112,082 cupin sequences matching to 6529 species and 945 related protein structures are listed [16]. More than 100 cupin sequences have been identified in few plant species like *Oryza sativa*, *Vitis vinifera* and *Arabidopsis thaliana*, this emphasizes the extent to which cupins have duplicated and diverged in proteomes of various organisms to carry out various functions [17]. Presence of short loops, elevated degree of sub-unit contacts and hydrophobic interactions are typical characteristics of cupin superfamily, which in-turn are responsible for its thermal stability, resistance to protease action and varying nature of oligomerization pattern [18, 19].

Proteins in the cupin superfamily have a wide range of biological functions in archaea, bacteria and eukaryotes. The functional classification of cupin superfamily is complicated, as diverse roles allocated to this superfamily is incessantly amplifying, at least 18 families embrace the diverse cupin superfamily of proteins, which include enzymatic functions like hydrolases, dioxygenases, decarboxylases, isomerases and epimerases non-enzymatic functions such as seed storage, binding to auxin, and nuclear transcription factors [15, 18].

Most cupin enzymes are metalloenzymes with two conserved sequences including the motifs [G(X)5HXH(X)3,4E(X)6G] and [G(X)5PXG(X)2H(X)3N] together with an 11 to 16-amino-acid inter-motif region [17]. It has been confirmed that the two His residues and the Glu residue in motif one together with the His residue in motif two act as ligands for the binding of an active-site metal ion, such as Fe, Mn, or Zn [20]. So far a protease comprising of cupin domain has never been reported. Cupincin as a protease is a new addition to the diverse cupin superfamily of proteins.

In the current study we have isolated and purified a protease from rice bran and named it as Cupincin, analysis of its sequence revealed that the protein belongs to cupin superfamily. This is the earliest report of a cupin domain containing protease. In addition, cupincin was biochemically characterised and its 3D structure build to understand its hydrolysing mechanism. Furthermore, cupincin was found to be a zinc metallo-protease and was specific in its action. These properties add considerable interest to an already important cupin superfamily of proteins and it would be fascinating to see whether the list would be added with more proteases or whether any other new enzymes will also be included in the diverse cupin superfamily of proteins.

## Materials and Methods

### Chemicals and Reagents

Commercially available, freshly milled rice bran (variety IR-64) purchased from Ganesh rice mills, Bannur (12.33°N 76.86°E), Mysuru district, Karnataka, India, was defatted by passing through hexane, air dried and sieved through mesh (No.18 BS) and stored in air tight container at –20°C for further use. 10 kDa molecular mass cut-off centrifugal device was purchased from Amersham Biosciences (Uppsala, Sweden). The dialysis tubing of 10 kDa molecular mass cut-off was procured from Spectrum, USA. Sephadex G-150 was purchased from Pharmacia (Uppsala Sweden). Oxidised B-chain of insulin, neurotensin and trypsin were purchased from Sigma. (7-methoxycoumarin-4-acetic acid- MCA)Leu-Val-Glu-Ala-Leu-Tyr-Leu-Val-Cys-Gly-Lys (Dinitrophenol- DNP) was synthesised from Biomatik, USA. Quartz triple distilled water was used for the entire analysis. Unless stated other chemicals used were of analytical/HPLC grade.

### Purification of cupincin

The crude enzyme is extracted from the flour using 50mM Tris-HCl buffer (pH-7.5) (1:5 w/v) by stirring for 90 minutes. The extract was centrifuged at 5485 g for 45 minutes. The (NH_4_)_2_SO_4_ concentration in the resulting supernatant was increased to 40% saturation and centrifuged at 5485 g for 40 minutes. The resulting supernatant obtained after 40% saturation was increased to 80% (NH_4_)_2_SO_4_ saturation and again centrifuged at 5485 g for 40 minutes. The precipitate thus obtained was dissolved in a minimum volume of Tris-HCl buffer and dialyzed exhaustively against 50mM Tris-HCl buffer (pH 7.5). The dialysate was lyophilised and stored at –20°C for further experiments. The concentrated solution of 80% (NH_4_)_2_SO_4_ saturation was applied onto a Sephadex G-150 column (0.45×80cm) and eluted with 50mM Tris-HCl buffer, pH 7.5. Fractions were eluted at a flow rate of 6mL/H and 2 mL fractions were collected. Alternate tubes of the collected fractions were assayed for protease activity as described later. Active fractions were pooled and concentrated using a centrifugal device (10kDa cut-off).

### Proteolytic activity

Proteolytic activity was assayed according to the procedure of Satake et al., [21], with modifications described. Haemoglobin (0.2 mL, 2% in 50 mM acetate buffer pH 4.0) was incubated with 0.1mL enzyme in a total volume of 0.5 mL for 60 min at 60°C. The reaction was stopped by adding 0.5 mL of 5% Tri chloro acetic acid (TCA); this mixture was allowed to stand for 30 minutes. In the case of blank, the substrate was added to the enzyme inactivated by TCA. The mixture was then centrifuged at 4535 g for 30 min and the resulting supernatant was again subjected to centrifugation at same conditions to remove any residual particles. Sodium carbonate (1.25 mL, 0.4 M) and 0.25 mL of Folin Ciocalteus reagent (diluted to 1:1 of the original strength in water) was added sequentially to 0.5 mL of the supernatant and the colour developed was read at 660 nm, after 30 minutes of incubation at room temperature. “One unit of enzyme activity is defined as the amount of the enzyme required to cause an increase in absorbance of 0.01 at 660nm/min at 60°C”.

### Effect of inhibitors on proteolytic activity

Purified cupincin was pre-incubated with 5mM solutions of EDTA, 2mM PMSF and iodoacetamide, 0.5mM of pepstatin A and 1mM of 1,10-phenanthroline for 15 minutes and proteolytic activity determined as described in the previous section. The enzyme activity without the above inhibitors was taken as 100% and the relative activities calculated with respect to it.

### Measurement of protein concentration

The protein concentration at different stages of purification was determined by measuring absorbance at 280 nm and by the dye-binding method of Bradford [22]. Bovine serum albumin (BSA) was used as the standard protein.

### Polyacrylamide gel electrophoresis (PAGE)

Sodium dodecyl sulfate Polyacrylamide gel electrophoresis (SDS-PAGE) was carried out using a discontinuous buffer system according to the method of Laemmli [23], using 10% gel (acrylamide (T) concentration 10% with bisacrylamide (C) crosslinker concentration of 2.7%). Electrophoresis was performed after denaturing the purified protease with sodium dodecyl sulphate (SDS) and β-mercaptoethanol. Native PAGE (10% T, 2.7%C) was performed at pH 8.8 in the absence of SDS and β-mercaptoethanol and the gels were stained with Coomassie brilliant blue R-250.

### Gelatin-embedded PAGE for protease assay

Zymographic analysis [24] was performed by incorporating gelatin 1% (w/v, final concentration) as the substrate to the polyacrylamide gel as described in the previous section for non-denaturing PAGE. Subsequently after electrophoresis at pH 8.8, the gel was washed several times with distilled water. After incubating the gel for 12 hours at 37°C in 50 mM sodium acetate buffer of pH 4.0, 0.1% Coomassie brilliant blue R-250 was used for staining the gel.

### N-terminal protein sequencing

N-terminal sequences of the subunits of cupincin were determined as described previously by Devaraj et al., [25] with modifications as follows, following SDS-PAGE cupincin subunits were transferred to polyvinylidene difluoride (PVDF) membrane in 20mM taubine-20% methanol buffer by electro-blotting at 0.8 A/cm^2^ at constant current for 90 minutes and stained with Coomassie brilliant blue R-250. N-terminal sequences of the sub-units were determined by automated Edman degradation using the gas-phase sequencer (Applied Biosystems 447A, Rotkreu, Switzerland).

### Electrofocusing

The isoelectric point of isolated enzyme was evaluated by analytical electro-focusing (Amersham Biosciences- Multiphor II, Uppsala, Sweden) in immobilized pH gradient containing ampholyne over the pH range 3.5–9.5. An isoelectric focusing calibration kit (Amersham Pharmacia Biotech), containing 11 proteins of known isoelectric points was used as a reference. The proteins were allowed to focus for 90 minutes at 1500 v.

### Analytical Gel filtration chromatography

The homogeneity and apparent molecular mass of the purified protease were determined by using Progel^™^-TSK W-3047 (7.88mm ID × 300mm) in HPLC system (Waters). The eluant used was 0.1 M sodium phosphate buffer pH 7.0 at a flow rate of 1 mL/min. The column was calibrated with Aprotinin (6.5 kDa), Cytochrome- C (12.4 kDa) Carbonic anhydrase (29 kDa), Bovine serum albumin (66 kDa) and -globulin (160 kDa).

### Determination of the molecular mass of cupincin using MALDI-TOF

The molecular mass of cupincin was determined by using a matrix-assisted laser desorption and ionization time of flight mass spectrometer (MALDI-TOF). The sample was dissolved in 50% acetonitrile containing 0.1% TFA. The sample solution was mixed with matrix solution (α-cyano-4-hydroxycinnamic acid) and loaded on a stainless steel MALDI target. Samples were measured in the reflection and positive ionization mode.

### Effect of pH on enzyme activity

The effect of pH on the enzyme activity of cupincin was evaluated from pH 2.0–7.0 using the following buffers: 50 mM Glycine-HCl (pH 2.0–3.0), 50 mM acetate buffer (pH-4.0, 5.0), 50 mM phosphate buffer (pH-6.0) and 50 mM Tris-HCl buffer (pH 7.0). The activity of the protease was determined at 60°C using denatured haemoglobin as the substrate.

### Effect of temperature on enzyme activity

The optimum temperature for activity was determined by incubating the enzyme in the temperature range of 30°C-70°C. Denatured haemoglobin (2%, w/v) in 50 mM acetate buffer, pH 4.0 was used as the substrate. The maximum activity obtained was taken as 100% and the relative activities are calculated with respect to it.

### Determination of the hydrolytic specificity

Hydrolytic specificity of cupincin was examined as described previously [25] with modifications, in which the oxidized B chain of bovine insulin and neurotensin were used as substrates for digestion and N-terminal sequences of the peptides formed were determined. The assay was carried out in acetate buffer pH 4.0 (50 mM) for different time intervals at 60°C. The purified enzyme to the substrate ratio was 4% (w/w, protein basis). 0.1% TFA was added to 40μL of the reaction mixture at different time intervals to terminate the reaction and it was further clarified by centrifugation. The digested peptides were fractionated by RP-HPLC using a C-18 small pore Grace Vydac column (4.6× 250mm, 5μM) on a Waters HPLC system. The peptides were detected at 230nm. The solvents used were 1% TFA and 70% acetonitrile containing 0.5% TFA. The peptides were identified by their amino terminal sequence following Edman degradation as described earlier.

### In-gel protein digestion

Protein bands on SDS-polyacrylamide gels were enzymatically digested in-gel as described previously [26] using modified trypsin. The resulting tryptic peptide fragments were extracted with 50% acetonitrile and 0.1% TFA with sonication.

### MS/MS analysis and data base search

Peptide sequences were identified as described previously [27]. Spectra obtained were processed using the flex analysis (Bruker Daltonics) software. The reflectron MS and MS/MS spectra were transferred to bio-tools and then submitted to National Center for Biotechnology Information non-redundant protein sequence databases (NCBInr) using the MASCOT search engine (http://www.matrixscience.com). Peptide mass tolerances used in the search were 50 ppm and fragment mass tolerance set was 0.5 Da.

### Structural analysis

#### Molecular modelling of cupincin

Schrodinger software was used for molecular modelling (Schrodinger Inc. U.S.A). The sequence for cupincin was taken from the database of NCBI [28] with accession id OsI_13867 that contains 436 amino acid residues with molecular weight 48492.94Daltons. The initial election of the 3SMH A chain structure as the template by the software was maintained. After modelling the structure was subsequently refined by energy minimization and evaluated by Schrodinger software. Homotrimeric structure of cupincin was generated using Swiss model [29–31]. Model quality was checked by using the Procheck server [32]. Cartoons were drawn using Chimera [33].

### *In silico* active site prediction by PMAP database

Putative active site residues were predicted in the modelled structure using PMAP database. The Proteolysis MAP (PMAP, http://www.proteolysis.org) is a user-friendly website intended to aid the scientific community in reasoning about proteolytic networks and pathways.

### Circular dichroism (CD) studies

CD measurements were made using a Jasco J-810 automatic spectropolarimeter fitted with a xenon lamp. The instrument was calibrated with d (+)-10-camphor-sulphonic acid ammonium salt. The lamp was purged continuously with nitrogen before and during the experiments. The scans were recorded thrice. The far-UV CD spectrum was recorded between 190 and 260 nm using a 1 mm path length cell and a protein concentration of 0.1 mg/ml in 50 mM Tris-HCl, pH 7.5. Secondary structure analysis was carried out using the CDSSTR method [34, 35] with reference database SP175 [36] available in DICHROWEB [35].

### Detection of metal ions

Cupincin in 50 mM tris-HCl (pH-7.5) was subjected to atomic absorption flame emission spectrophotometer (Shimadzu AA-6701 F). The samples were filtered through 0.2m filter membrane before detection. A sample of buffer alone was also run on the instrument as a control. The elements Cu, Fe, Mn, Zn and Cd in the sample were analysed by Atomic absorption spectroscopy (AAS). The results were compared to a calibration curve in the range of 0.1–0.4 ppm Zn^2+^.

### Kinetic analysis of cupincin

Kinetic analysis was measured using a quenched fluorescence assay, with a synthetic peptide substrate based on the cupincin’s cleavage site (MCA)Leu-Val-Glu-Ala-Leu-Tyr-Leu-Val- Cys-Gly-Lys(DNP) (Biomatik, USA). Enzyme reactions were carried out at 60°C, in brown microfuge tubes, in a final volume of 400 μL of reaction buffer (50 mM sodium acetate, pH 4.0), containing varying concentrations of substrate (40–120μM) dissolved completely in Dimethyl sulfoxide (DMSO), and 50 μg (0.369 μM) of cupincin. All reactions were stopped by boiling samples at 100°C for 10 min, followed by centrifugation at 4535g for 30 min, 50 μL of reaction sample was removed and diluted in 500 μL of deionised water and fluorescent 7-methoxycoumarin-4-acetic acid (MCA) cleavage product measured. An excitation wavelength of 323 nm and an emission wavelength of 382 nm were used to measure the hydrolysis of the substrate on a Shimadzu RF 5301 PC fluorescence spectrofluorometer, and readings compared against a standard curve of MCA, dissolved in DMSO. Kinetic parameters *K*_*m*_ *and V*_*max*_ were calculated using Microsoft Excel graphing software.

## Results

### Purification of cupincin

**Fig 1:**
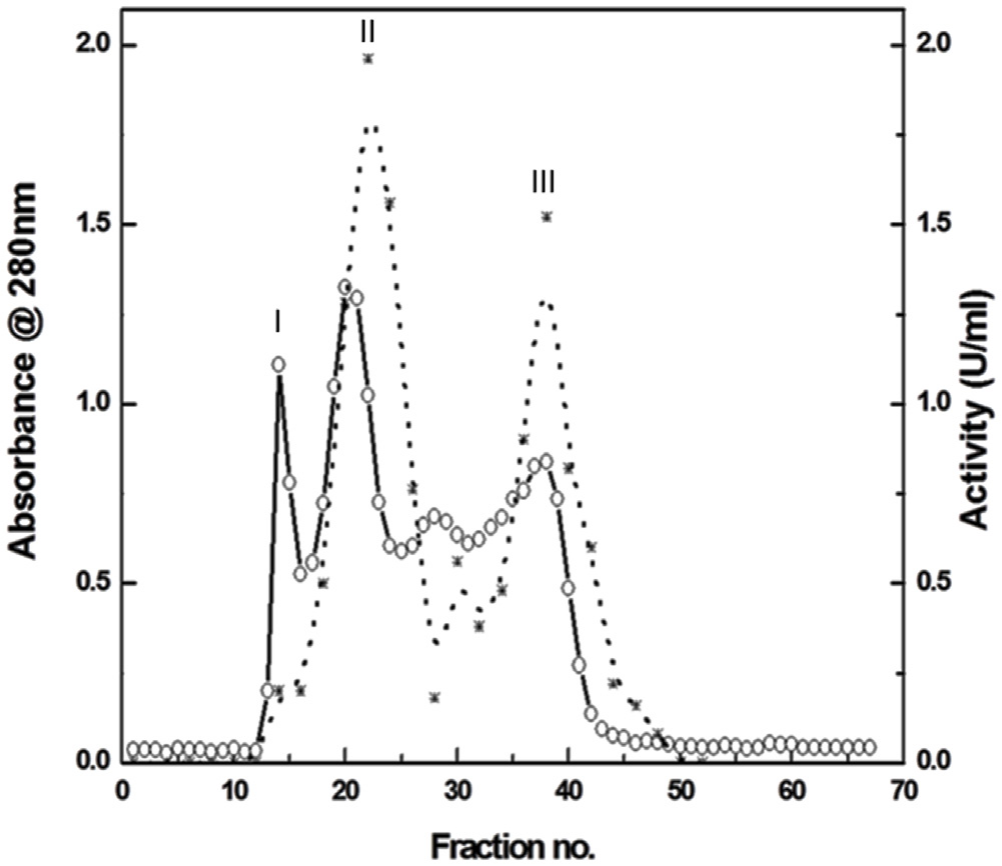
Size exclusion chromatography profile of cupincin on sephadex G 150 column. (o-o) protein content (*-*) protease activity. The column was pre equilibrated with 50mM Tris-HCl buffer, pH 7.5. A flow rate of 1mL/10 min was set. Fractions were detected at 280 nm. Peak II being highest in terms of proteolytic activity is pooled, concentrated and used for further experiments as described in the materials and methods section. Denatured haemoglobin was used as substrate for activity measurements.

The crude extract from rice bran was precipitated with ammonium sulphate (0–40%). Ammonium sulphate concentration in the resulting supernatant was raised from 40% to 80% saturation as the second step of purification. The first step of 40% saturation facilitates to concentrate the enzyme to a workable volume that could be further subjected to 80% saturation. The lyophilized 80% precipitated rice bran protein sample (30 mg) was fractionated using Sephadex G-150 column, followed by appropriate elution; the resulting profile is resolved into three peaks (Fig 1), peak II being the highest in magnitude in terms of both proteolytic activity and homogeneity. The majority of the total activity loaded on the column was eluted in this peak from which the active fractions eluted were pooled (Fig 1) and concentrated using a centrifugal device (10 kDa cut-off). Analysis of these fractions gave total protein, total protease activity and specific activity as shown in the Table 1. The enzyme was purified to homogeneity with 4.4 folds increase in specific activity (1.78 units/mg) having a yield of 4.9%. The purification protocol and the yields, as well as specific activity of the enzyme preparation were consistent.

**Table 1:** Purification of cupincin from rice bran*.

Steps	Total activity (units)	Total protein (mg)	Specific activity(units/mg)	Purification (Fold)	Recovery (%)
Crude	713.4	1771.2	0.40	1	100
80% (NH_4_)_2_SO_4_ precipitation	411.25	384.88	1.06	2.65	21.72
Sephadex G-150 size exclusion chromatography	155.06	86.77	1.78	4.43	4.9

* These are typical purification results from 50 g of rice bran. These values are reproduced in three separate purifications. Denatured haemoglobin was used as the substrate for activity measurements.

### Characterization of cupincin

**Fig 2:**
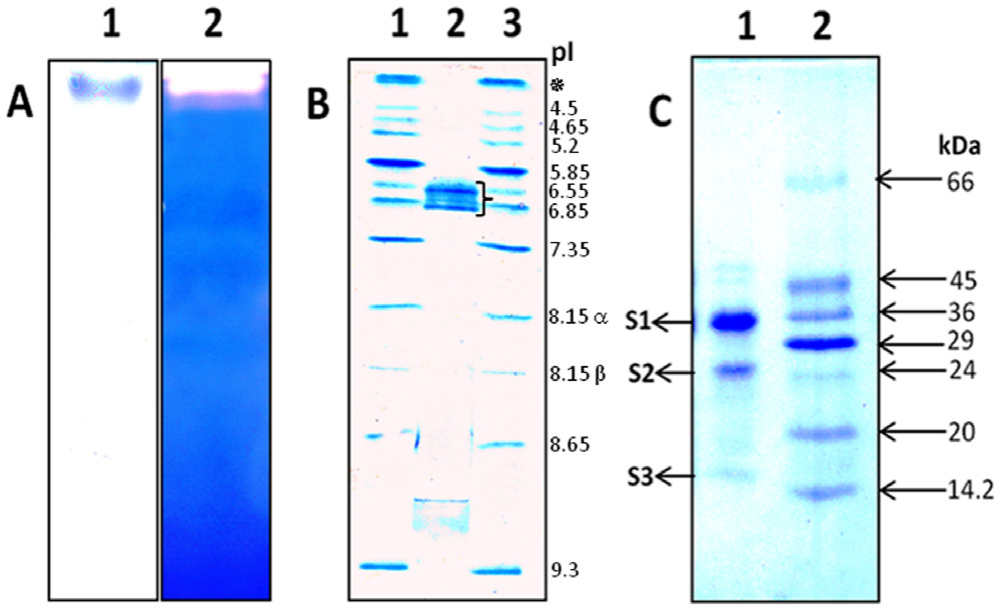
Biochemical characterisation of cupincin. **A)** Lane 1: Native PAGE (10% T, 2.7% C) of the purified cupincin. Lane 2: Gelatin-embedded PAGE for detecting protease activity stained with coomassie brilliant blue. (**B)** Iso electric focusing: Lane 1: Broad range pI calibration kit (pI- 3 to10, ✵-amyloglucosidase-coloured pI marker, 8.15α-lentil lectin-acidic band, 8.15β-lentil lectin-middle band). Lane 2: Iso electric point of cupincin was determined to be 6.6–6.8, while focussing in the pI range of 3–9, flower bracket indicating the cupincin bands, Lane 3: Broad range pI calibration kit (pI 3–10). (**C)**. SDS-PAGE (10%T, 2.7% C) of purified cupincin, Lane 1: Purified cupincin, Lane 2: M_r_ Markers.

The homogeneity of cupincin was assessed by analytical gel filtration, native-PAGE and gelatin embedded zymography. The purified enzyme showed a single band in the native conditions (Fig 2A, Lane 1). In gelatin embedded PAGE, the presence of protease was detected as a clear white band against a dark blue background due to the hydrolysis of gelatin (Fig 2A, Lane 2). In native conditions the enzyme was not able to migrate into the resolving gel, since it has an iso-electric point of 6.6–6.8, focusing in a pI range of 3–9 (Fig 2B). However in SDS-PAGE, the purified enzyme showed three sub-units at 34.48±.94 kDa, 26.87 kDa and 15.93±0.46 kDa (Fig 2C). Estimation was based on the calibration curve constructed with known molecular mass markers (6.5–66 kDa) (S1 Fig).

**Fig 3:**
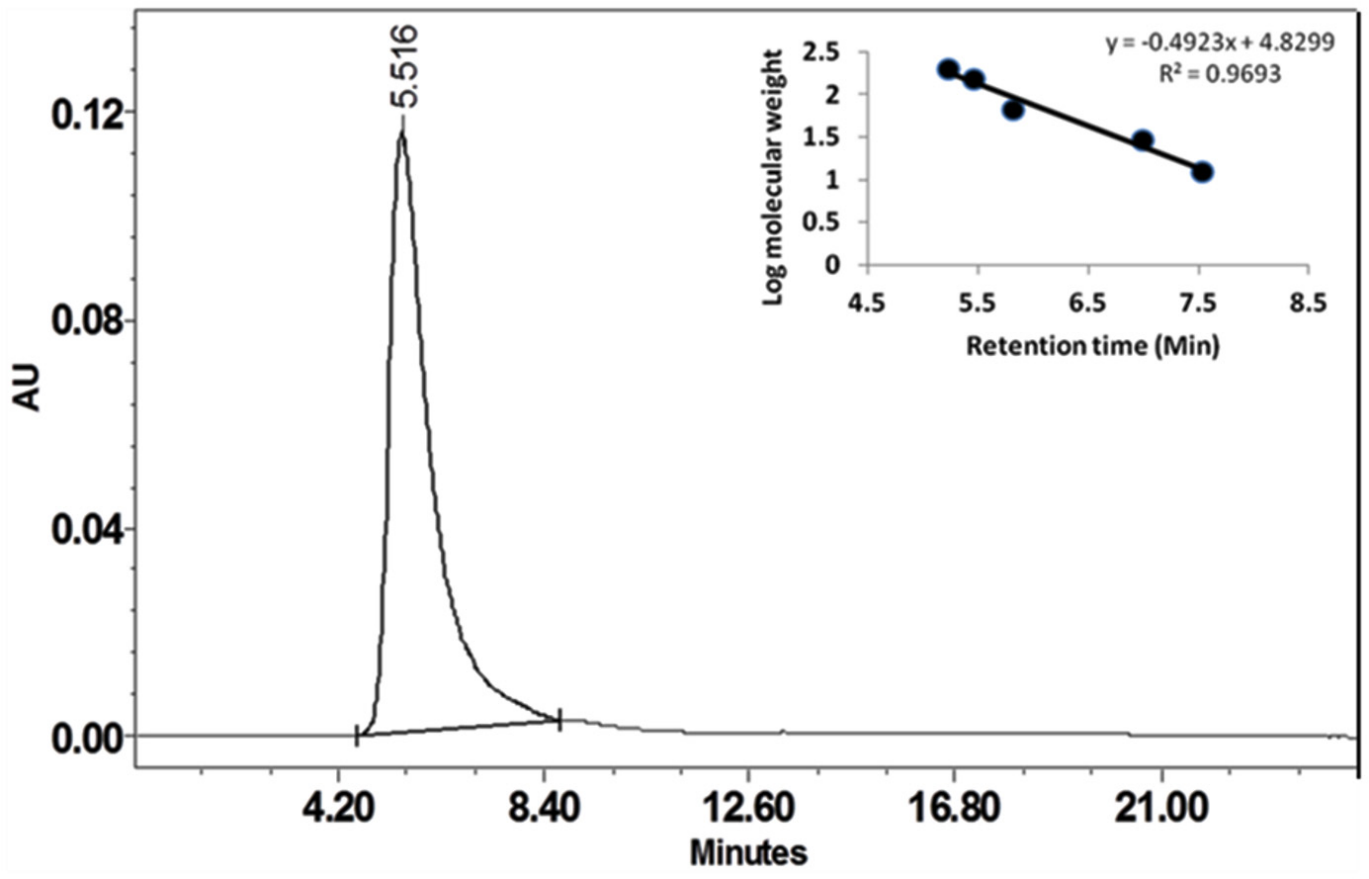
Elution profile of cupincin on analytical gel filtration column. Assessment of the molecular mass of cupincin. x-axis- retention time in minutes, y-axis- Absorbance unit (Au) @ 280ηm. The column was calibrated with 1) ϒ-Globulin, 2) Bovine serum albumin, 3) Carbonic anhydrase, 4) Cytochrome C and 5) Aprotinin. The column was equilibrated with 0.1 M sodium phosphate buffer pH 7.0 and the proteins were eluted at a flow rate of 1 mL /min. The samples were run for 25 minutes. Retention time of cupincin was determined to be 5.516 minutes corresponding to the mass of around 135 kDa as estimated based on the calibration curve constructed with known molecular mass proteins in the range of 6.5kDa-160 kDa (inset).

**Fig 4:**
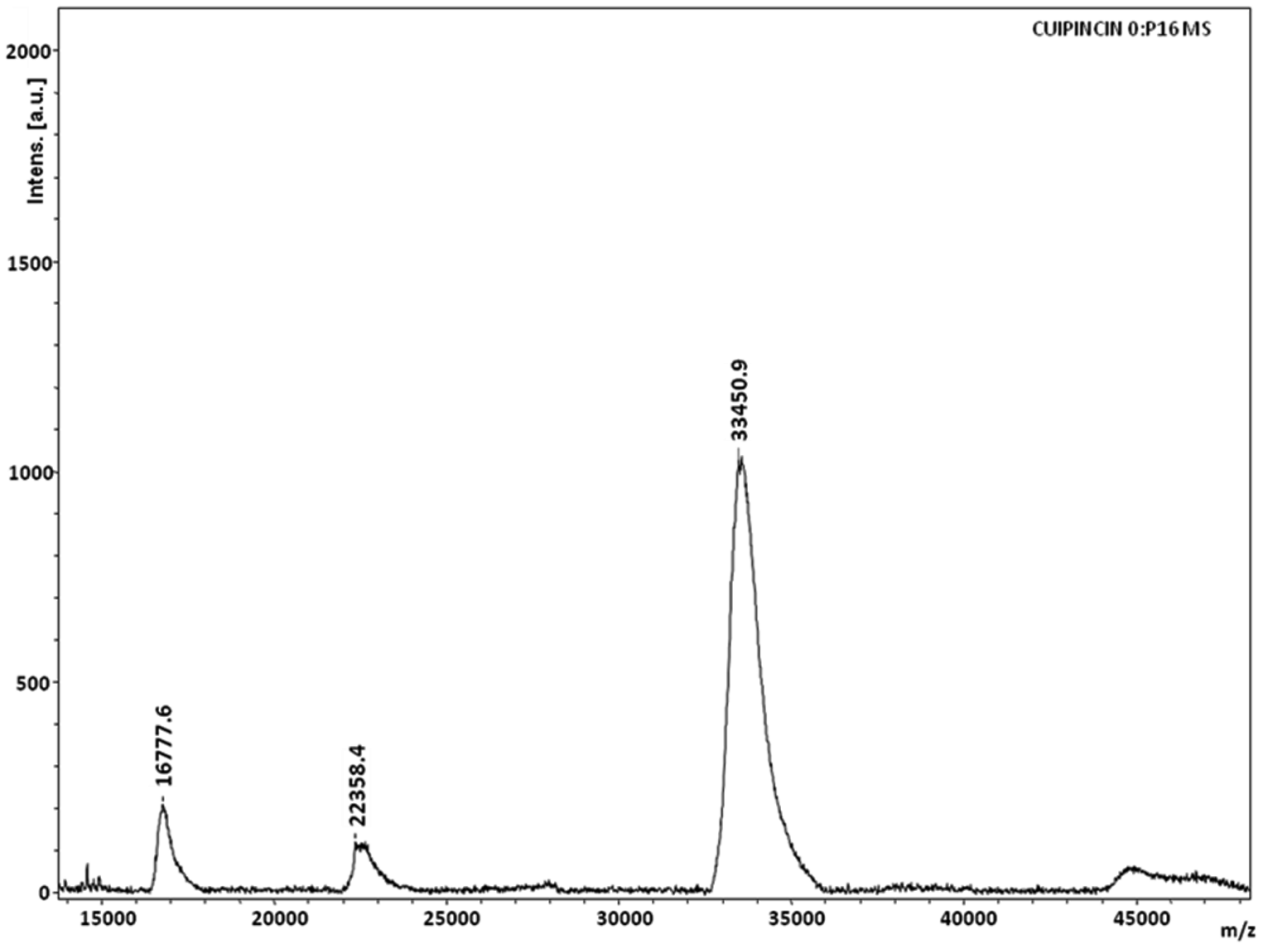
MALDI-TOF spectrum of cupincin. Protein sample was mixed in equal volume 1:1 with matrix Sinapinic acid 50% Acetonitrile and water with 0.1% TFA and spotted on the target plate and air dried. MS spectrum was obtained using UltrafleXtreme, Bruker Daltonics spectrometer operating in linear and positive ionization mode with Flex control software.

**Fig 5:**
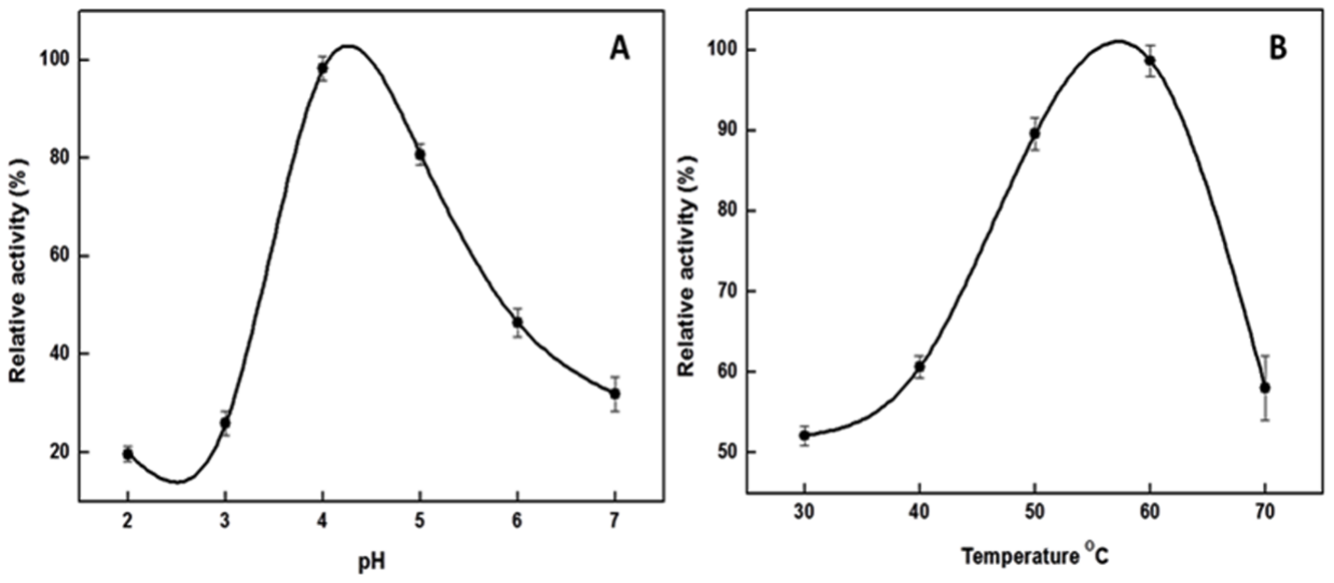
**(A).** Optimum pH (●-●) of purified protease from rice bran. Activity measurements were carried at 60°C, buffers used are specified in the methods. (**B).** Optimum temperature (●-●) for purified protease activity measurements were carried at pH 4.0. Respective temperatures were maintained ±1°C using a water bath. Values were expressed as mean ± SD (n = 3).

Cupincin eluted as a single peak after molecular sieve chromatography on Progel^™^-TSK W-3047 column by HPLC (Fig 3). The apparent molecular mass of the purified protease was found to be 135.33 ± 3.5 kDa, using standard proteins in the range of 6.5 kDa-160 kDa (Fig 3, inset). The result of MALDI-TOF spectra (Fig 4) corresponds well with the results of mass determined in SDS-PAGE and analytical gel filtration, confirming its homogeneity and its homotrimeric nature. The effect of pH on the enzyme activity of purified protease was evaluated from pH 2.0–7.0 (Fig 5A) cupincin had an optimum pH of 4.0, the enzyme is relatively active at lower pH, however beyond pH 5.0 there is a sharp decline in the activity. The optimum temperature for activity was determined by incubating the enzyme in the temperature ranging from 30°C-70°C (Fig 5B). The optimum temperature of cupincin was found to be 60°C, the enzyme retained 60% of its original activity even at 70°C, which indicates its thermal stability.

### Hydrolytic specificity of cupincin

**Fig 6:**
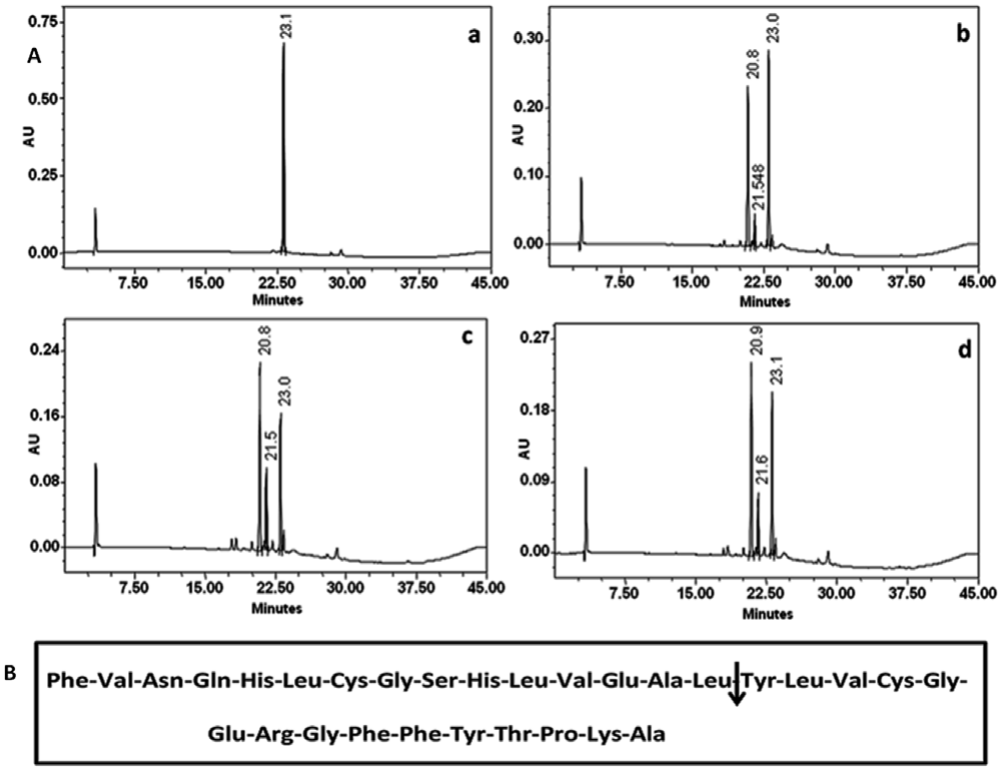
Hydrolytic specificity of cupincin on B-chain of insulin. **(A).** RP-HPLC profile showing the blank Insulin B chain (a). Cleavage pattern of B chain of insulin hydrolyzed by cupincin at 1hour (b), 2hours (b) and 3 hours (d) of incubation. The peptides were resolved using a Grace Vydac C-18 column (4.6 × 250mm). The solvents used were 0.1% Trifluoroacetic acid (TFA) and 70% acetonitrile containing 0.05% TFA. The peptides were detected at 230 nm. **(B).** Amino acid sequence of B-chain of insulin, arrow indicating the cleavage position of cupincin.

**Fig 7:**
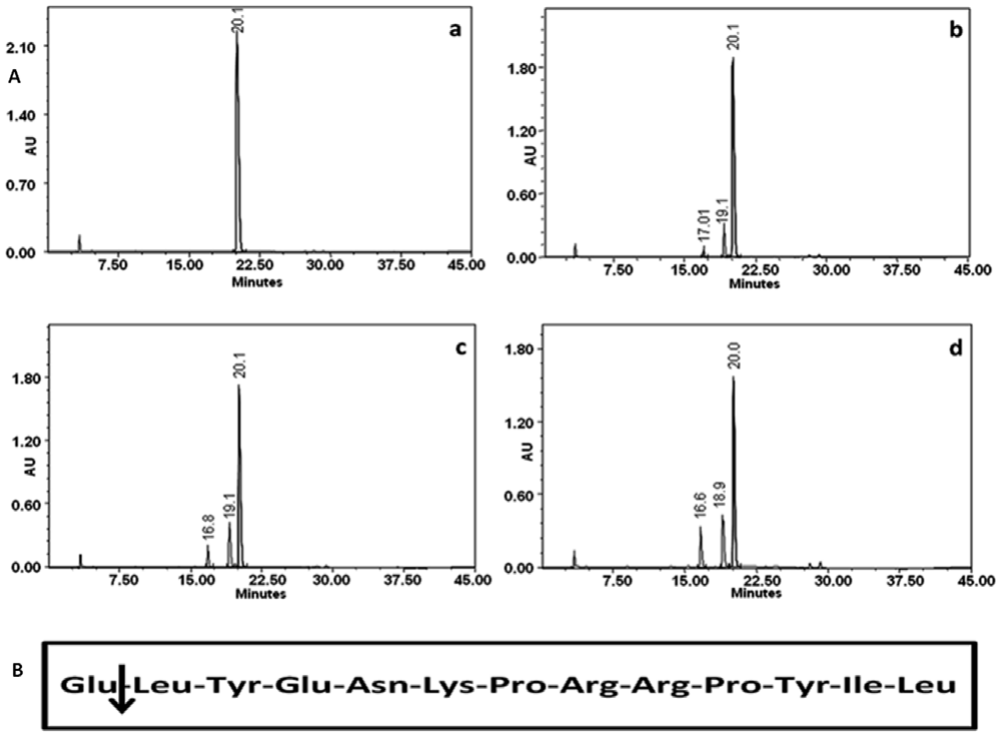
Hydrolytic specificity of cupincin on neurotensin. **(A).** RP-HPLC profile showing the blank neurotensin (a). Cleavage pattern of neurotensin hydrolyzed by cupincin at 2hours (b), 4hours (c) and 6 hours (d) of incubation. The peptides were resolved using a Grace Vydac C-18 column (4.6 × 250mm). The solvents used were 0.1% TFA and 70% acetonitrile containing 0.05% TFA. The peptides were detected at 230 nm. **(B).** Amino acid sequence of neurotensin, arrow indicating the cleavage position of cupincin.

The cleaved peptides from oxidised B chain of insulin and neurotensin were resolved by RP-HPLC as shown in Figs 6 and 7. The results showed that only three peptides were formed after 1–6 hours of digestion. With longer hydrolysis time, the substrate peak decreased, the concentration of the newly formed peptides increased, but no additional peptides were formed. N-terminal amino acid sequence determination of the peptides indicated that only one peptide bond was cleaved by cupincin, the Leu_15_-Tyr_16_ peptide bond in B-chain of insulin and Glu_1_-Tyr_2_ position in neurotensin.

### Effect of protease inhibitors on cupincin activity

Relative activity of cupincin in the presence of group-specific protease inhibitors was carried out to determine the nature of the protease (Table 2). 1mM of 1, 10-phenanthroline a high-affinity zinc chelator inhibited 84% of enzyme activity. However, EDTA and other class-specific protease inhibitors did not show any effect on the enzyme activity.

**Table 2:** Effect of inhibitors on the activity of cupincin.

Inhibitor	Concentration (mM)	Relative activity (%)
EDTA	5	100%
PMSF	2	100%
Iodoacetamide	2	96.7%
Pepstatin A	0.5	91.3%
1,10-Phenanthroline	1	16%

### Atomic absorption spectroscopy

Atomic absorption spectroscopy (AAS) analysis of cupincin showed that only Zn^2+^ was present, occupying ~26.52% of the cupincin molecules. None of the other elements, Cu, Fe, Mn, Cr, or Cd, were detected.

### Kinetic parameters of cupincin

K_m_ and K_cat_ values of Cupincin were found to be 123.869 μM and 0.0531min^-1^ respectively using synthesised fluorogenic peptide which was synthesised based on the cleavage preference of cupincin (S2A & S2B Fig).

### Structural characterization of cupincin

**Fig 8:**
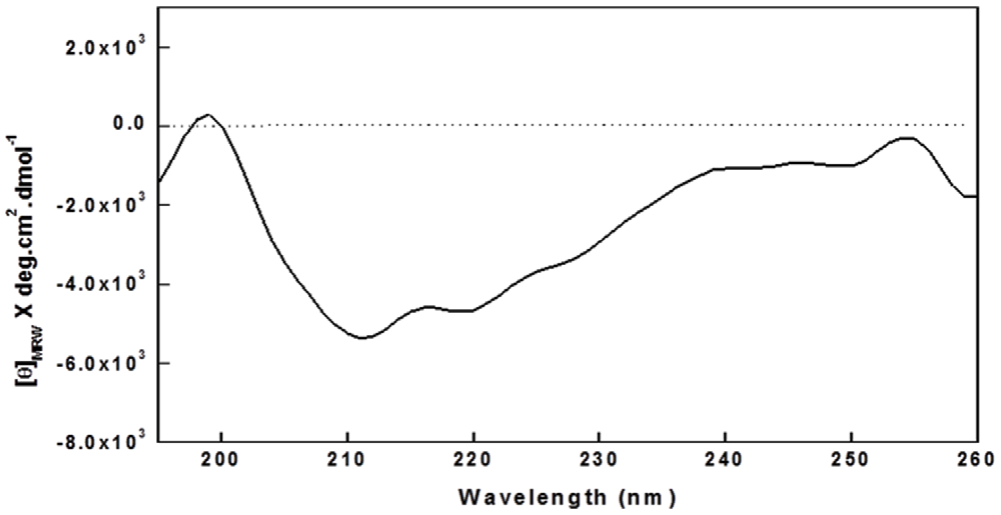
Far UV CD spectra of cupincin. 0.1 mg/ml of protein in 50 mM Tris-HCl, pH 7.5. Accumulations of three runs are shown.

The far UV CD spectrum of cupincin is shown in Fig 8. The spectrum shows a broad minimum in the region of 210-218nm, indicative of a predominant β structure. The CDSSTR method, using SP175 as the reference database, gave the best fit and was used to estimate secondary structure content. This method revealed 51.1% β structure, 17.8% α helix and 31.1% aperiodic structure in the protein, indicating the protein belongs to cupin superfamily.

**Fig 9:**
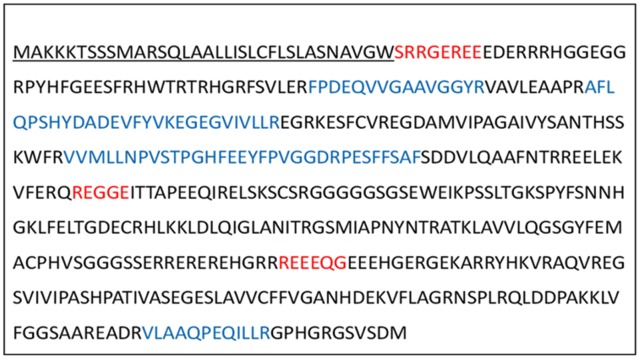
Deduced amino acid sequence of cupincin. The putative signal sequence is underlined (S6 Fig). Amino terminal sequence as determined by Edman degradation method is indicated in red. Peptide sequences obtained by in-gel trypsin digestion and MS/MS analysis are indicated in blue (S8A–S8G Figs). OsI_13867 (NCBI) or LOC Os06g43830.1 (TIGR data base, http://rice.plantbiology.msu.edu/) was identified as cupincin.

**Fig 10:**
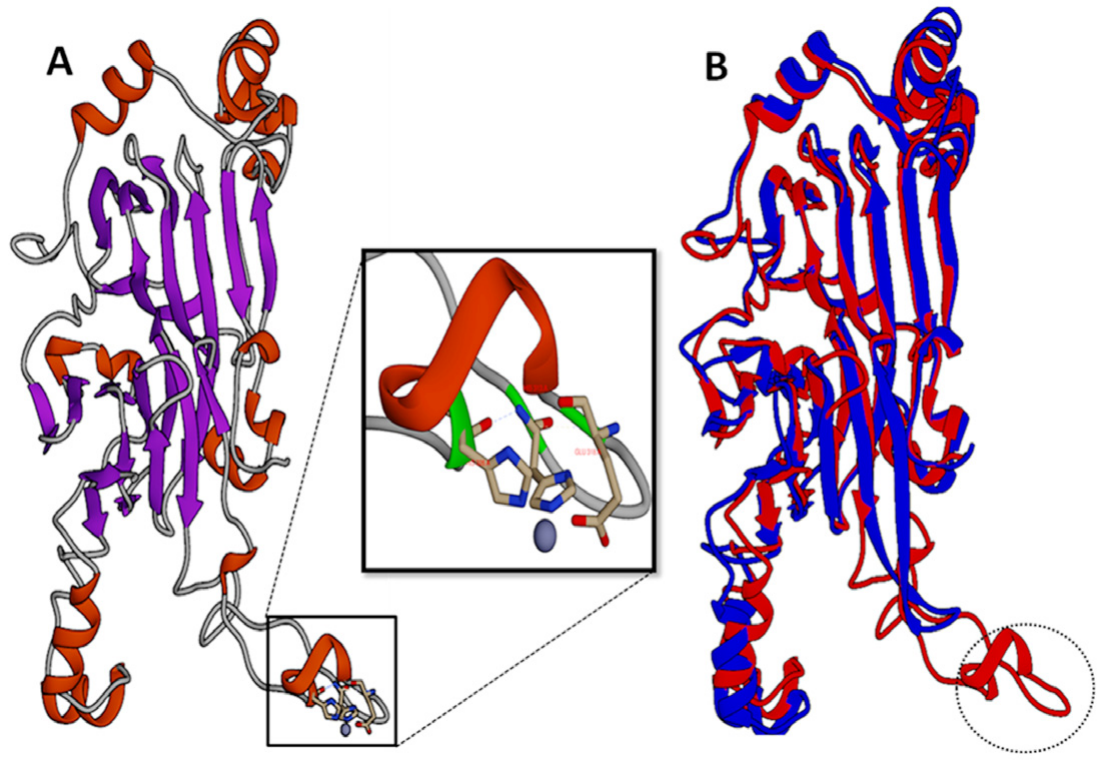
3D modelled momomeric structure of cupincin. **(A)**: Illustration to show the computed monomeric structure of cupincin containing typical characteristics of cupin superfamily. Alpha helix portions are coloured in red, pleated beta sheets in blue. The beta-rich cupin fold can be easily distinguished between the alpha-helix segments that contribute to inter-subunit interactions. **Fig 10A (Magnified):** View of the active site of cupincin the zinc ion (dark grey ball) coordinated by conserved protein residues His 313, HIS 326 and GLU 318. (**B):** Superimposition of homology modelled cupincin structure (Red) with its template 3SMH A chain (Blue). (http://www.dsimb.inserm.fr/dsimb_tools/ipba/index.php). Broken circle indicating the presence of active site region only in cupincin but missing in the template.

**Fig 11:**
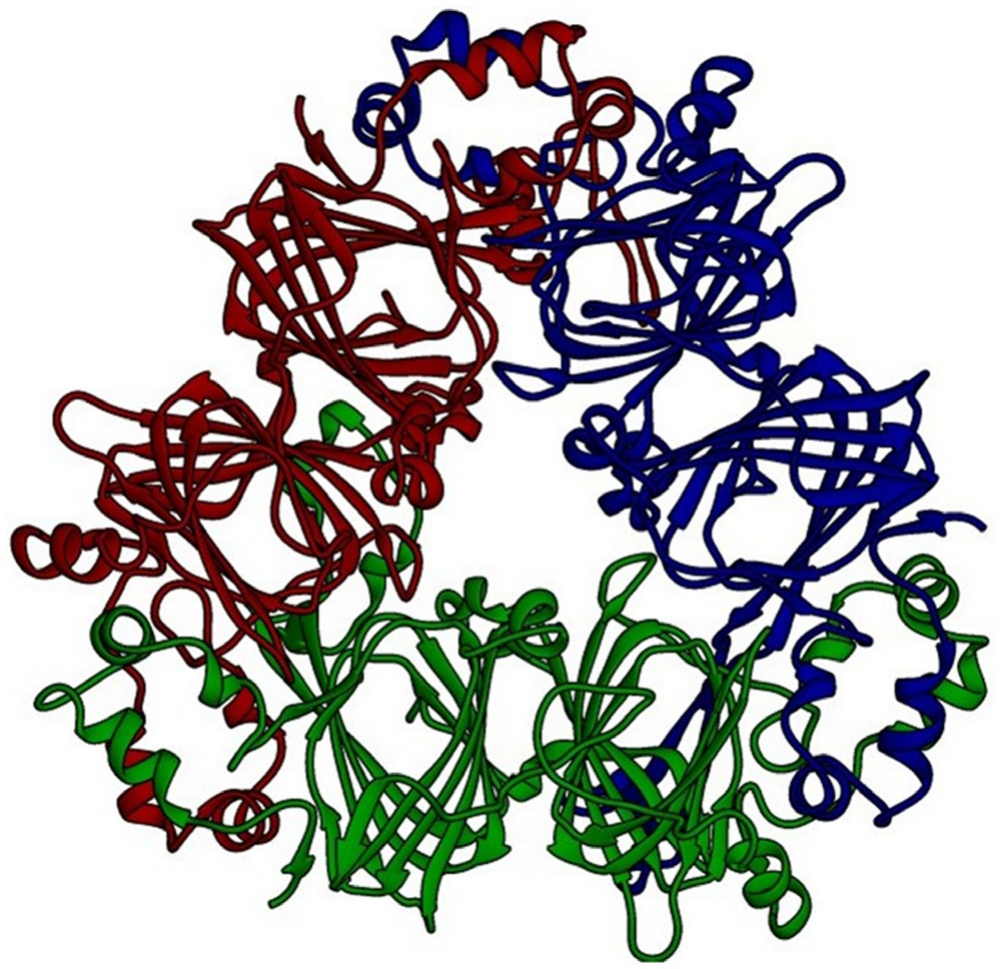
3D modelled trimeric structure of cupincin. View of the trimeric structure of cupincin as modelled in Swiss-Model (The Swiss Institute of Bioinformatics) [29–31]. Each subunit of the trimer is coloured differently (http://swissmodel.expasy.org/workspace/).

OsI _13867 (NCBI) annotated as hypothetical protein was identified as cupincin by deducing its amino-terminal sequence and *de-novo* sequencing, the N-terminal sequences of three subunits separated in SDS-PAGE S1, S2 and S3 (Fig 2C) were identified to be SRRGEREE, REGGE and REEEQ respectively (Fig 9, indicated in red). A three-dimensional model of cupincin was build *in-silico* to predict the structure-function relationship and to propose a functional model to validate our experimental findings. Structural analysis revealed cupincin to be a homotrimer (Figs 10 & 11), containing His313, His326 and Glu318 with a zinc ion at the active site (Fig 10 magnified and S9 Fig). Superimposition of homology modelled cupincin structure with its template 3SMH A chain indicated that the presence of catalytic triad only in cupincin, however missing in the template (Fig 10B).

**Fig 12:**
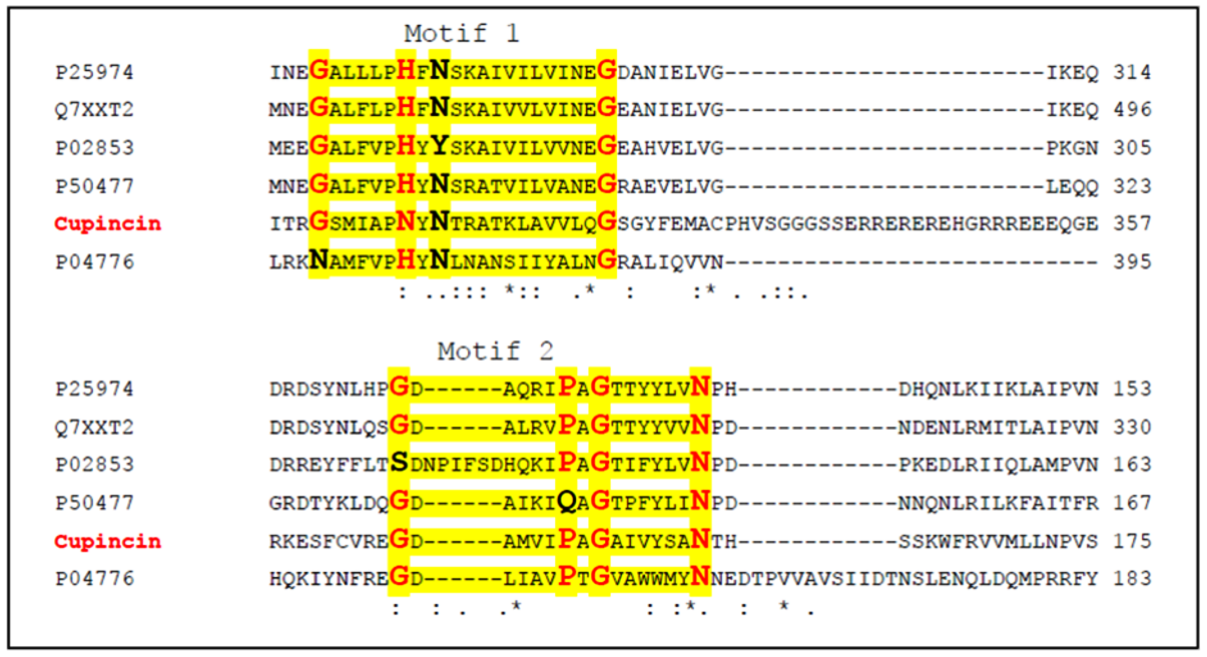
Cupincin as bicupin. Multiple sequence alignment of cupincin with trimeric seed storage proteins (bicupins) showing two conserved domains (highlighted in yellow with consensus sequence indicated in bold red). The sequences utilised for alignment are as follows. P25974 [50], P50477 [51], P02853 [52], P04776 [61], Q7XXT2 [62].

From database search, cupincin was confirmed to be a member of the cupin superfamily (S3A and S3B Fig) and it was found to be a bicupin (Figs 10 & 12). Apart from *Oryza sativa*, cupincin shares 67% sequence identity with TPA: globulin2 from *Zea mays* (DAA51858.1), 42% identity with globulin from *Sesamum indicum* (AAK15089.1) and *Elaeis guineensis* (AAK28402.1) (S4 Fig). Sequence comparisons failed to reveal any similarity with any other known proteases, establishing cupincin as a completely new class of proteolytic enzyme.

## Discussion

In the current study we have isolated and purified a protease comprising of cupin domain for the first time. Cupincin was purified to homogeneity by simple procedures of ammonium sulphate fractionation and size exclusion chromatography and was found to be a zinc metallo protease with specific cleavage preference. Although numerous proteins of cupin superfamily have been structurally characterized, the functions of many of them have not been experimentally determined. The first two-domain proteins recognized to be members of the cupin superfamily were the seed storage proteins [37]. Cupins are found in a wide range of cell types and have a wide range of biochemical functions. In both pro and eukaryotes, these proteins are consistently associated with stress response. In higher plants, seed storage proteins (SSP’s) are allied with desiccation tolerance, typically SSP’s are specialised group of proteins containing at most a single conserved histidine and lacking any enzymatic activity [37].

According to the primary sequence of cupincin, it is associated with nutrient reservoir activity (OsI_13867, NCBI). It is from the current study we can recruit a specific proteolytic activity to this so far uncharacterized protein. The results of molecular modelling, N-terminal sequencing, MALDI-TOF, SDS-PAGE and analytical gel filtration revealed that the protein is a homotrimer with three distinct subunits. The three polypeptide bands of cupincin S1, S2 and S3 were subjected to N-terminal amino acid sequencing and compared with the sequence of OsI_13867. The N-terminal sequencing of the main components in cupincin revealed that the 36.91, 28.57, and 13.07 kDa polypeptides contained amino acid sequences that matched three regions of the sequence of OsI_13867 annotated in NCBI. The major polypeptide band of 36.91 kDa (S1), is a duplet comprising of the 21.42 and 15.5 kDa polypeptides and the 28.57 kDa polypeptide (S2), comprised of the 15.5 and 13.07 kDa polypeptides (Calculation of mass of subunits is according to the primary amino acid sequence determined from the commencement of three N-terminal sequences of each subunit in cupincin). This result suggests that the polypeptides S1, S2 and S3 correspond to OsI_13867. The differences in molecular weight of the three bands probably arose from different cleavage sites from the C-terminus. The total mass of the protein according to the sequence of OsI_13867 would be 48.5 kDa, whereas the native mass of the protein was determined to be 135.33 ± 3.52 kDa in analytical gel filtration. Molecular modelling suggested cupincin as a homotrimer, thus the sequence mass of cupincin of 48.5 kDa as homotrimer would be in accordance with the native mass of purified protein. The result of MALDI-TOF spectra corresponds well with the results of mass determined in SDS-PAGE and analytical gel filtration, confirming its sub-unit stoichiometry and homotrimeric nature.

Cupincin’s specificity on oxidized B chain of insulin and neurotensin is much higher than any other plant proteases reported. However an aspartate protease associated with wheat gluten was also shown to preferentially cleave only the Leu_15_-Tyr_16_ peptide bond of oxidized B chain of insulin [38]. This high selectivity of cupincin on oxidized B-chain of insulin and neurotensin indicates that the enzyme has more specific function other than mobilization of storage proteins, may be like processing enzymes as described for aspartate proteases from barley and members of the *Brassicaceae* [38]. The reason for altered specificity for cupincin on neurotensin, may be due to the bond for preferential cleavage (L-Y) is at the opening position in neurotensin, thereby hindering its proper binding to the substrate. The susceptibility of peptide bonds to proteolysis are determined by the sequence in the vicinity of the scissile bond and the way in which the bond is displayed in terms of its structural context [39]. Specific binding of proteases not only depend on the nature of catalytic centre, but also due to the series of binding sites favouring particular amino acids on either side of the catalytic centre. This intensity of preciseness ensures that proteases carry out their task only when truly needed. Therefore to be effective, proteases must interact precisely with their substrates and bind firmly to them [40].

Limited proteolysis is essential for the initiation of storage protein breakdown. The proteolysis of seed storage protein for providing plants with amino acids during early seedling growth is a step-wise process; limited nature of initial proteolysis steps is due to initiating enzymes having strong preference for certain cleavage sites in native storage proteins. The structural changes induced by the initial cleavages of the storage proteins result in conformation changes that open them to further degradation by other protease present in the same compartment [41]. Rapid limited mobilization of a susceptible part of protein reserves prior to their massive degradation might be a simple explanation for the functional role of storage globulin limited proteolysis [42]. Shutov and Vaintraub [43] hypothesised that limited proteolysis initiates profound hydrolysis of storage globulin molecules and thus it is a prerequisite for their massive degradation. Shutov et al., [44] demonstrated the probable structural alterations of storage 11S globulin from soybean seeds (glycinin) molecules by limited proteolysis, which in turn regulates the subsequent massive degradation of glycinin by one-by-one proteolyses mechanism. The alterations of the primary and higher order structures of glycinin generated by limited proteolysis were also tentatively analyzed in that study. This mechanism of limited proteolysis prohibits cleavage by proteinases that are simultaneously present in the same compartment [45].

In monocots, storage protein mobilisation in plant storage vacuoles involves acidification of the starchy endosperm [46, 47]. In maturing seeds pH for regulating enzyme activity has been ascribed to be between pH 3.0 and 5.5, with an optimum at pH 4.5 [48].This acidification produces an environment conducive to protease activity and also increases the solubility of SSPs [46,48,49]. Optimum conditions of cupincin at acidic conditions and its specific amino acid preference for cleavage hints its role as an initiating endopeptidase. But the exact mechanism of action of cupincin is the challenge to be solved in the near future.

The *K*_*m*_ *and K*_*cat*_ of cupincin were found to be 123.869 μM and 0.0531min^-1^ respectively. However the synthesised peptide was only soluble completely in DMSO since the reactions were not carried at the optimum conditions of cupincin (pH-4.0), the calculated *K*_*m*_ *and K*_*cat*_ of cupincin would be inexact.

The estimated content of secondary structure of cupincin was consistent with that obtained by theoretical modelling with a large predominance of β-strand structure (51.1%) over the -helix (17.8%). These values are consistent with the secondary structure content of several members of the bicupin group of vicilin-like proteins with known three-dimensional structures, such as b-conglycinin (14.1% helix, 36.7% β -sheet; [50], canavalin (11.0% -helix, 36.9% β -sheet; [51] and phaseolin (16.6% -helix, 36.0% β -sheet; [52].

According to the number of cupin domains in their structure cupins may be grouped into monocupins, bicupins, and multicupins, [53, 54]. From a structural point of view, members of seed storage proteins contain two cupin domains and are therefore, classified as bicupins [53, 54]. In bicupins, the two domains are structurally similar while the sequence similarity can be extremely low [18]. According to 3D modelling cupincin was determined to be homotrimeric bicupin. The final 3D model shows the typical characteristics of storage globulins in particular of the cupin superfamily. Individual subunit of the homotrimer has two central cupin domains created majorly by anti-parallel beta-sheets flanked by zones rich in alpha-helix. Such domains in the centre of the subunit are involved in the interaction between the subunits.

Proteins with identical superfamily can be assertively inferred to have evolved from a common ancestor, even if they might or may not posses any enzymatic activity [55–57]. Submission of the deduced sequence of cupincin to *MEROPS* (merops.sanger.ac.uk) database, a comprehensive data base of proteases and inhibitors and Protident [(http://www.csbio.sjtu.edu.cn/bioinf/Protease/), (S5 Fig)] showed negative results, however submission of modelled 3D structure of cupincin to PMAP database [58] (http://www.proteolysis.org) which combines five databases (ProteaseDB, SubstrateDB, CutDB, ProfileDB and PathwayDB) and a computational toolkit to create an integrated reasoning environment to analyze proteolytic networks indicated that cupincin was indeed a protease with His313, His326 and Glu318 as the catalytic triad (S9 Fig).

Cupins are usually metalloproteins in which the metal binding amino acid residues of cupin domain accommodates array of metal ions [17]. A characteristic of cupin superfamily proteins is that they contain a metal ion in their active sites. In most cupin domain containing proteins, metal ion is iron, but copper, zinc, nickel, cobalt, and manganese are occasionally present [20]. 3-His-1-Glu metal coordination pattern is clearly most typical of members of the cupin superfamily, although variations on this theme have been reported [59]. Cupincin was inhibited by 1,10 phenanthroline indicating that cupincin is a metalloprotease. PDB site scan [60] predicted that there is a zinc metal ion in active site of cupincin. Atomic absorption spectroscopy (AAS) analysis showed that only Zn^2+^ was present, occupying ~26.52% of the cupincin molecules. None of the other elements, Cu, Fe, Mn, Cr, or Cd, were detected. Therefore, cupincin is probably a metalloenzyme containing a Zn ion in the active site. Novel storage proteins are often reported to posses multiple roles like having enzymatic activity. The globulin storage proteins were basically deactivated enzymes from which the metal binding ligands had been lost during the course of evolution [19]. Its a matter of debate whether cupincin had retained its enzymatic activity by its ability to bind to zinc. This is the first biochemical characterization of a protease with cupin fold, which indeed represents a novel type of this enzyme. Further studies on this protease will shed more light in defining the enzyme mechanism in more detail.

In summary we have isolated and purified a novel cupin domain containing metallo protease from rice bran and OsI_13867 was identified as cupincin. Cupincin was exceedingly precise in cleavage of peptides suggesting it to have a definite function in rice. In the current study characterization of cupincin has revealed the diverse role of cupin domain. It will be interesting to further investigate the amino acids in the putative catalytic domain of cupincin to be able to postulate a new reaction mechanism for this new member of cupin superfamily. Further studies on this protease, involving cloning, expression and crystallization are currently underway.

## Supporting Information

S1 Fig Determination of molecular mass of cupincin by SDS-PAGE. Standard molecular mass markers used were in the range of 6.5 kDa-66 kDa.
(TIFF)

S2 Fig **(A)**: Fluorescence emission spectra of 7-methoxycoumarin-4-acetic acid (MCA) at different concentrations (0–30μM) were measured at an excitation wavelength of 323 nm and an emission wavelength of 382 nm. **(B)**: Lineweaver-Burk plot for action of Cupincin. [s] is expressed in μM; v is μM of MCA liberated per minute. Reactions were carried out at 60°C, containing varying concentrations of substrate (40–120μM), and 50 μg (0.369 μM) of cupincin.
(TIF)

S3 Fig **(A):** Sequence search results of “Cupincin” in Pfam database (http://pfam.sanger.ac.uk/search/sequence/results/58e33329-180c-4a3d-9921-9e0319875d67). **(B):** Graphic summary of “Cupincin” in NCBI (http://www.ncbi.nlm.nih.gov).
(TIF)

S4 Fig NCBI Blast search results of Cupincin. (TIFF)

S5 Fig Prediction of protease type in ProtIdent server (http://www.csbio.sjtu.edu.cn/bioinf/Protease/). (TIFF)

S6 Fig Prediction of signal peptide cleavage site using signal-P server (www.cbs.dtu.dk/services/SignalP/). (TIFF)

S7 Fig Calibration curve for Zinc ion in atomic absorption spectroscopy in the range of 0.1–0.4 ppm. (TIFF)

S8 Fig **(A):** Evidence for Internal sequence “FPDEQVVGAAVGGY”**(B):** Evidence for Internal sequence “NPVSTPG” **(C):** Evidence for Internal sequence “AFLQPSHYDADEVFYV” **(D):** Evidence for Internal sequence “FPDEQVVGAAVGGY” **-(E):** Evidence for Internal sequence “VVMLLNPVSTPGHFEEYFNGGDRPES” **(F):** Evidence for Internal sequence “VLAAQPEQILLR” **(G):** Evidence for Internal sequence “EGEGVIVLL”.
(TIF)

S9 Fig Prediction of putative active site residues in cupincin using PMAP database (http://www.proteolysis.org). (TIF)
