# Supplementary figures and images for: Cupincin: A Unique Protease Purified from Rice (Oryza sativa L.) Bran Is a New Member of the Cupin Superfamily
Source: PLoS One. 2016 Apr 11;11(4):e0152819. doi: 10.1371/journal.pone.0152819 (PMC4827828; doi:10.1371/journal.pone.0152819)

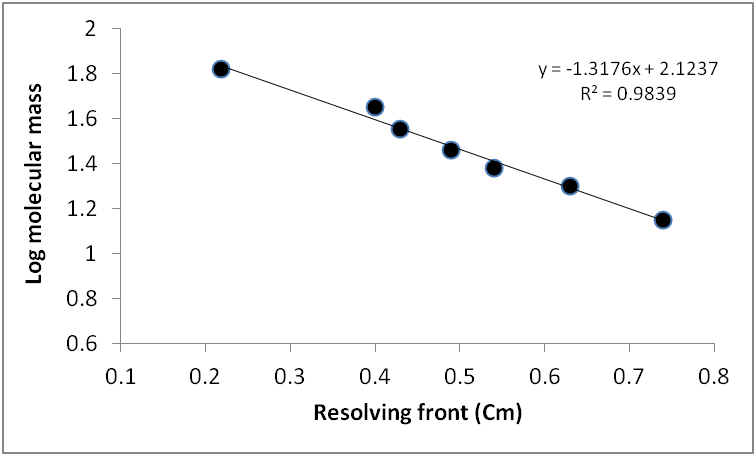

Supplement: S1 Fig — Standard molecular mass markers used were in the range of 6.5 kDa-66 kDa. (TIFF) [file pone.0152819.s001.tiff]

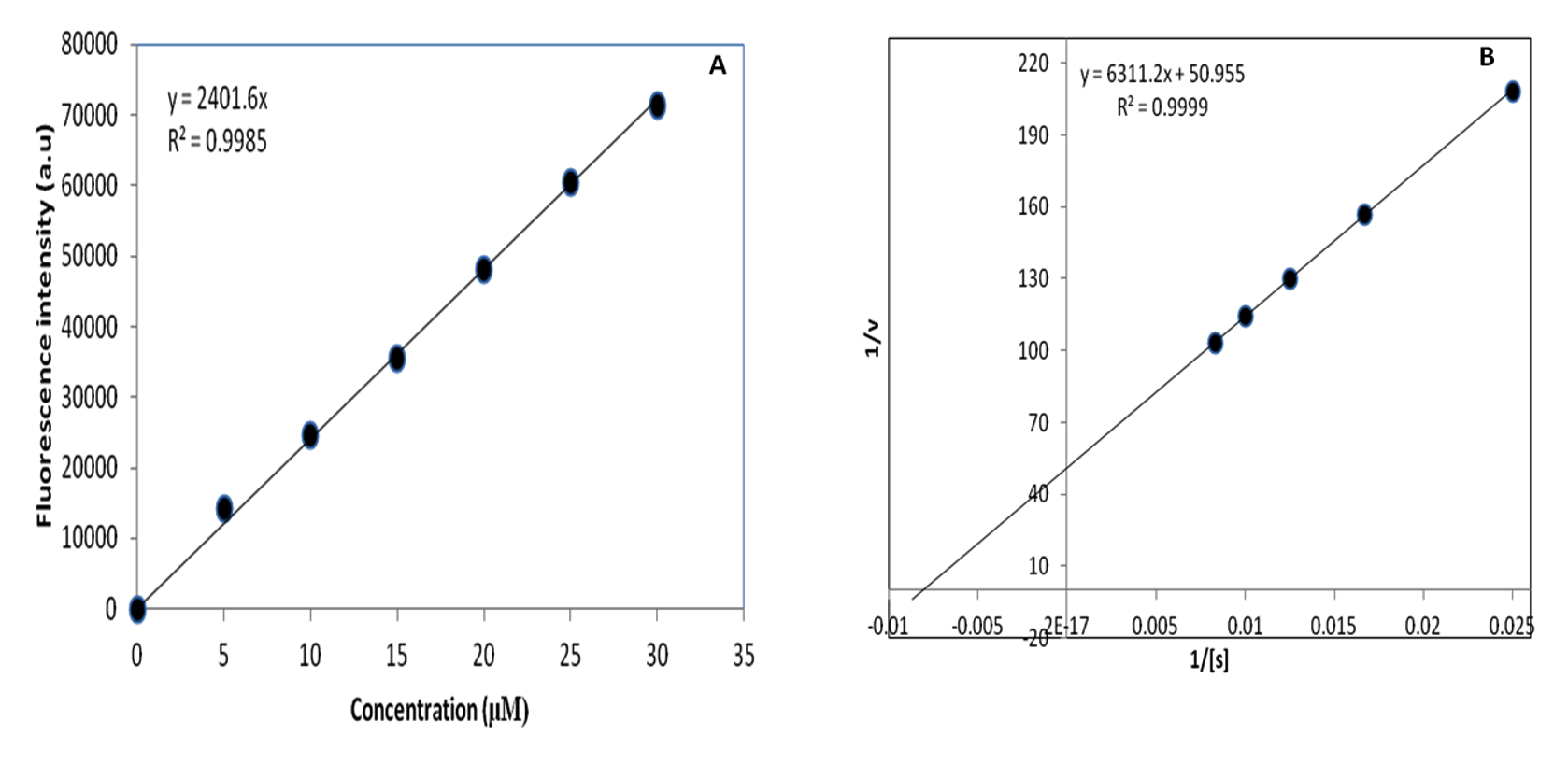

Supplement: S2 Fig — (A): Fluorescence emission spectra of 7-methoxycoumarin-4-acetic acid (MCA) at different concentrations (0–30μM) were measured at an excitation wavelength of 323 nm and an emission wavelength of 382 nm. (B): Lineweaver-Burk plot for action of Cupincin. [s] is expressed in μM; v is μM of MCA liberated per minute. Reactions were carried out at 60°C, containing varying concentrations of substrate (40–120μM), and 50 μg (0.369 μM) of cupincin. (TIF) [file pone.0152819.s002.tif]

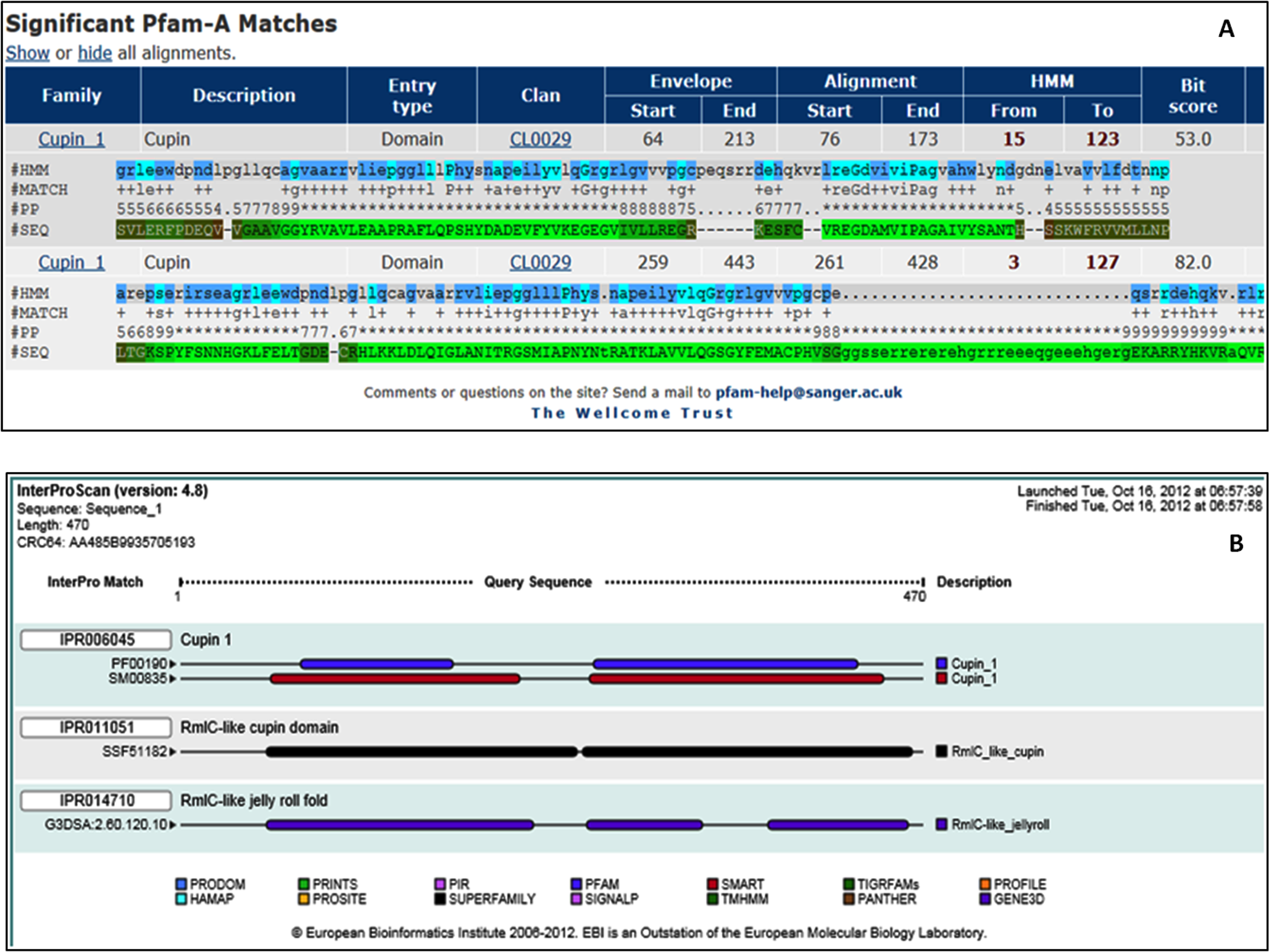

Supplement: S3 Fig — (A): Sequence search results of “Cupincin” in Pfam database (http://pfam.sanger.ac.uk/search/sequence/results/58e33329-180c-4a3d-9921-9e0319875d67). (B): Graphic summary of “Cupincin” in NCBI (http://www.ncbi.nlm.nih.gov). (TIF) [file pone.0152819.s003.tif]

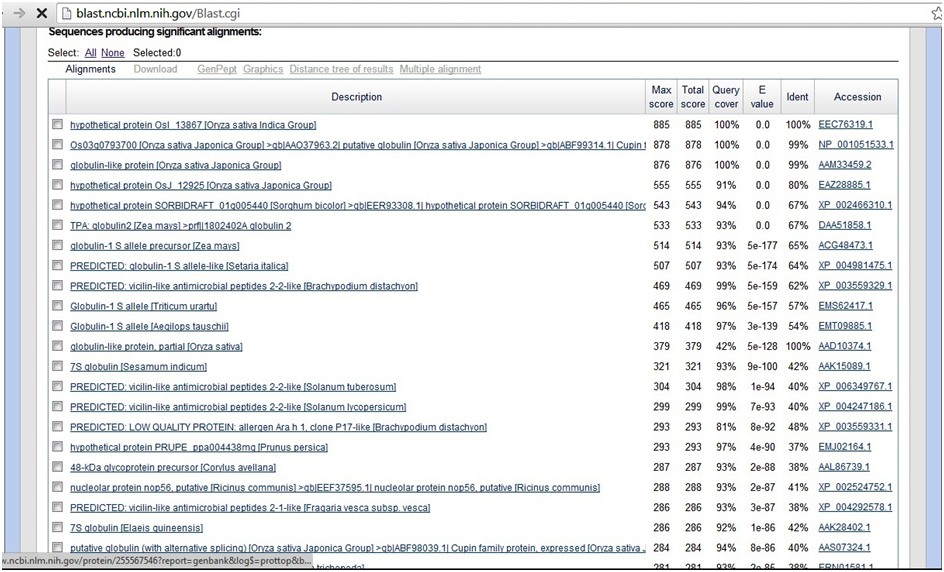

Supplement: S4 Fig — (TIFF) [file pone.0152819.s004.tiff]

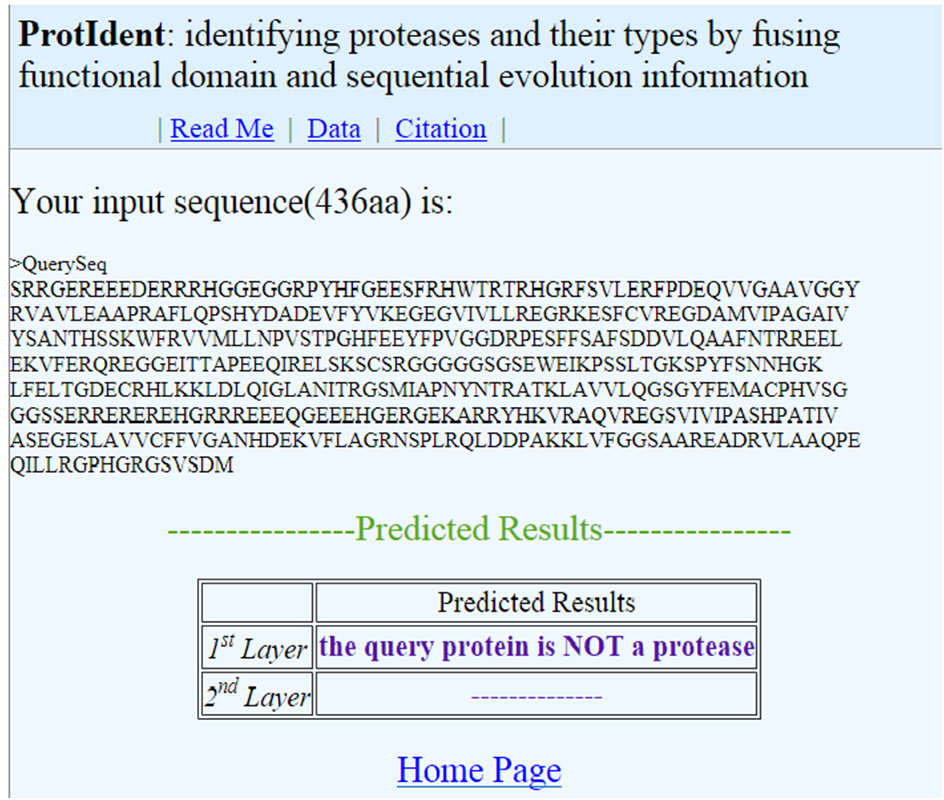

Supplement: S5 Fig — (TIFF) [file pone.0152819.s005.tiff]

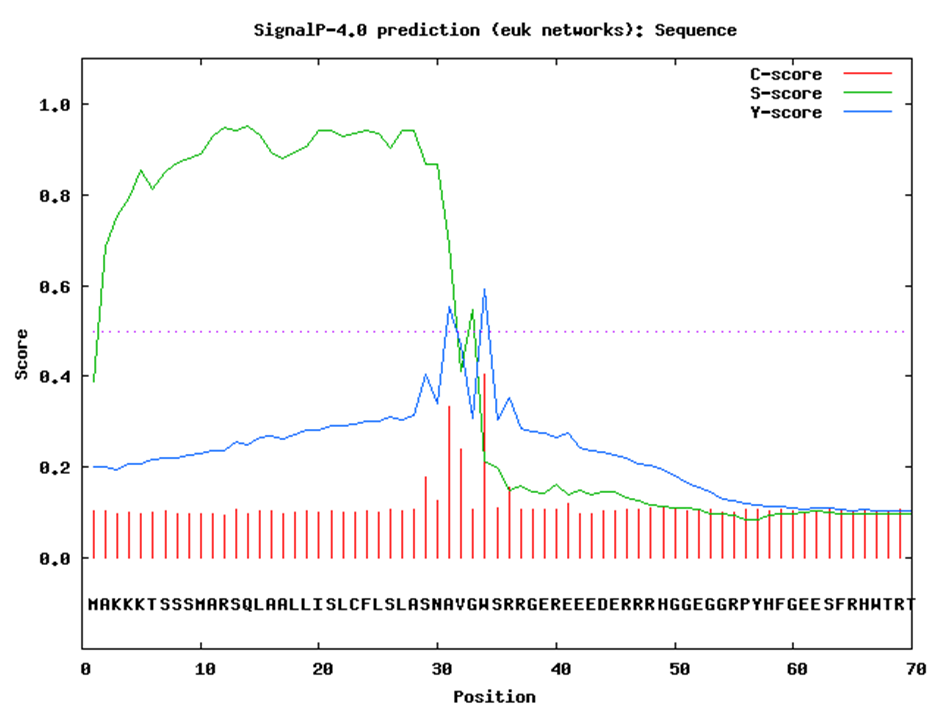

Supplement: S6 Fig — (TIFF) [file pone.0152819.s006.tiff]

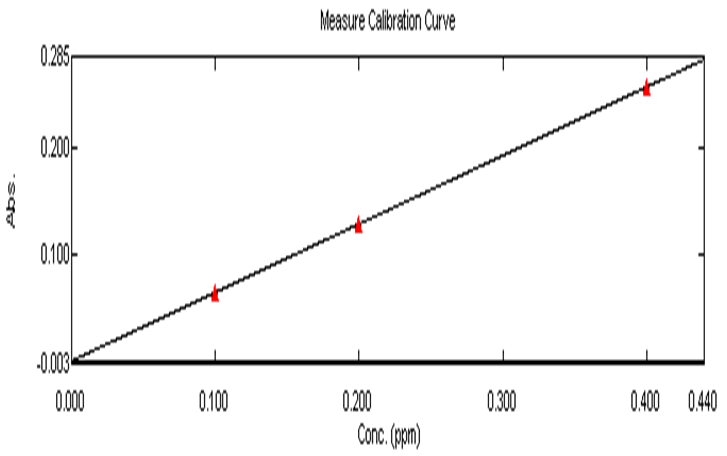

Supplement: S7 Fig — (TIFF) [file pone.0152819.s007.tiff]

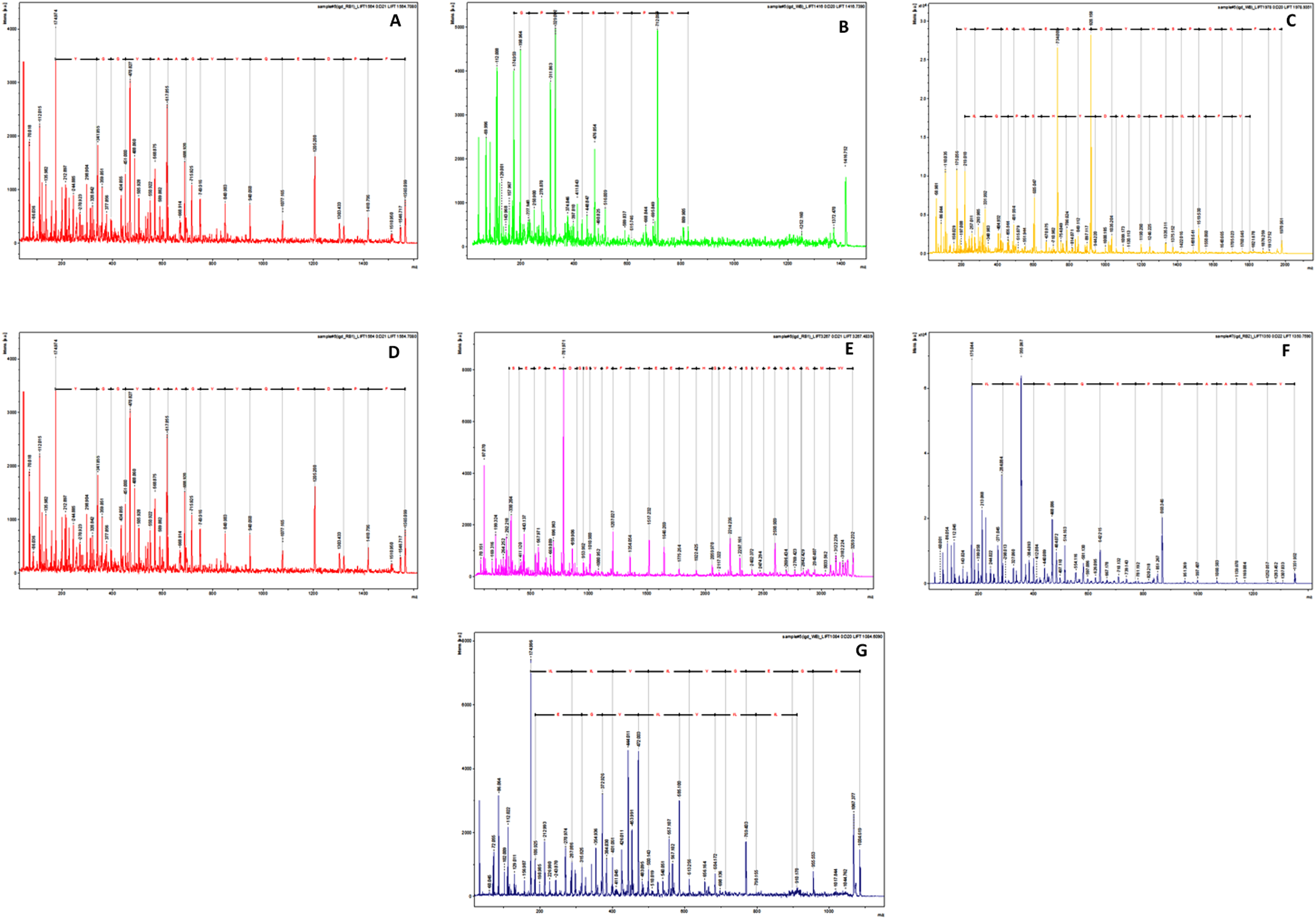

Supplement: S8 Fig — (A): Evidence for Internal sequence “FPDEQVVGAAVGGY”(B): Evidence for Internal sequence “NPVSTPG” (C): Evidence for Internal sequence “AFLQPSHYDADEVFYV” (D): Evidence for Internal sequence “FPDEQVVGAAVGGY” -(E): Evidence for Internal sequence “VVMLLNPVSTPGHFEEYFNGGDRPES” (F): Evidence for Internal sequence “VLAAQPEQILLR” (G): Evidence for Internal sequence “EGEGVIVLL”. (TIF) [file pone.0152819.s008.tif]

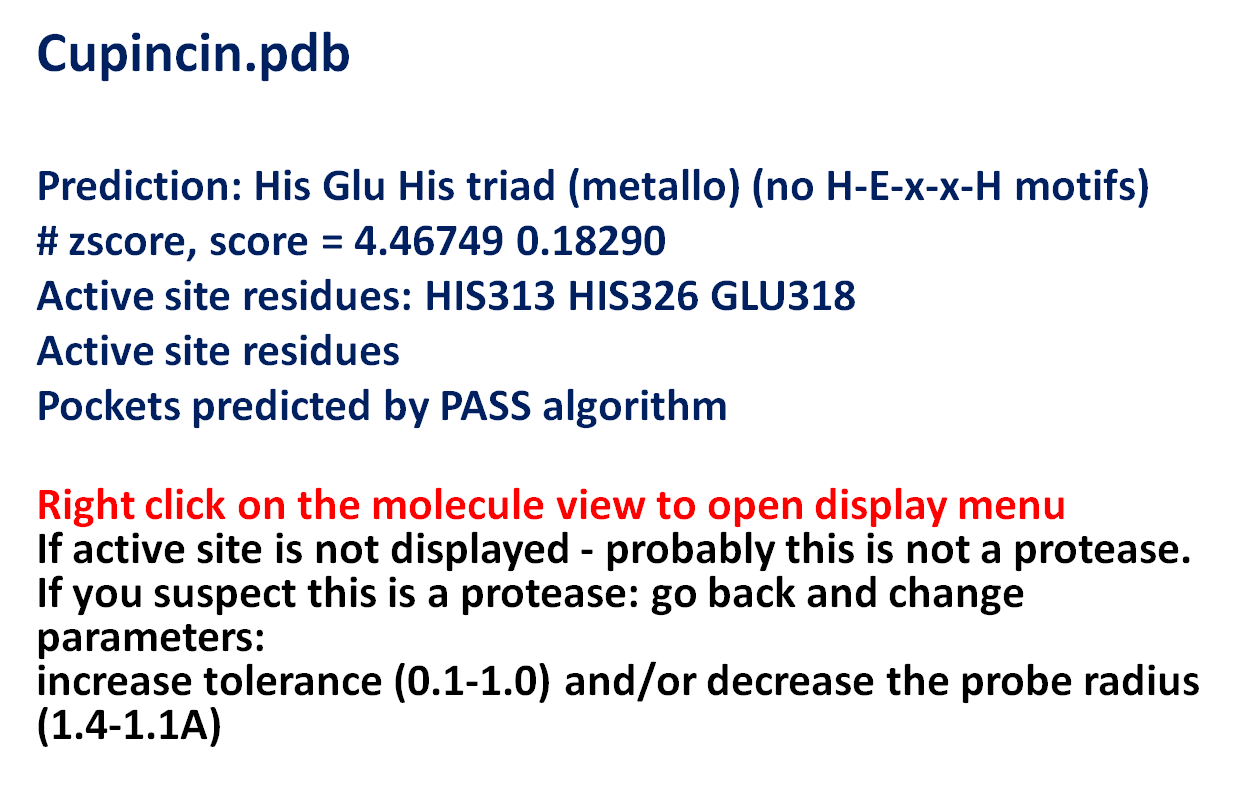

Supplement: S9 Fig — (TIF) [file pone.0152819.s009.tif]
